# Case report: Reversible brain atrophy with low titer anti-amphiphysin antibodies related to gastric adenocarcinoma

**DOI:** 10.3389/fneur.2023.1211814

**Published:** 2023-06-21

**Authors:** Ryota Amano, Yeon-Jeong Kim, Toshikazu Yoshida, Makoto Hara, Hideto Nakajima, Toshihisa Ohtsuka, Masanobu Yazawa

**Affiliations:** ^1^Department of Neurology, Fujimi-Kogen Hospital, Fujimi-Kogen Medical Center, Nagano, Japan; ^2^Department of Biochemistry, Graduate School of Medical Sciences, University of Yamanashi, Yamanashi, Japan; ^3^Division of Neurology, Department of Medicine, Nihon University School of Medicine, Tokyo, Japan

**Keywords:** anti-amphiphysin syndrome, paraneoplastic neurological syndrome, brain atrophy, immunoprecipitation, neuropathy, encephalopathy

## Abstract

Amphiphysin (AMPH) autoimmunity is associated with a variety of neurological complications, including encephalitis, peripheral neuropathy, myelopathy, and cerebellar syndrome. Its diagnosis is based on clinical neurological deficits and the presence of serum anti-AMPH antibodies. Active immunotherapy, such as intravenous immunoglobulins, steroids, and other immunosuppressive therapies, has been reported to be effective in most patients. However, the extent of recovery varies depending on the case. Herein, we report the case of a 75-year-old woman with semi-rapidly progressive systemic tremors, visual hallucinations, and irritability. Upon hospitalization, she developed a mild fever and cognitive impairment. Brain magnetic resonance imaging (MRI) showed semi-rapidly progressive diffuse cerebral atrophy (DCA) over 3 months, while no clear abnormal intensities were observed. The nerve conduction study revealed sensory and motor neuropathy in the limbs. The fixed tissue-based assay (TBA) failed to detect antineuronal antibodies; however, based on commercial immunoblots, the presence of anti-AMPH antibodies was suspected. Therefore, serum immunoprecipitation was performed, which confirmed the presence of anti-AMPH antibodies. The patient also had gastric adenocarcinoma. High-dose methylprednisolone, and intravenous immunoglobulin were administered and tumor resection was performed, resulting in resolution of the cognitive impairment and improvement in the DCA on the post-treatment MRI. After immunotherapy and tumor resection, the patient's serum was analyzed using immunoprecipitation, which showed a decrease in the level of anti-AMPH antibodies. This case is noteworthy because the DCA showed improvement after immunotherapy and tumor resection. Additionally, this case demonstrates that negative TBA with positive commercial immunoblots do not necessarily indicate false positive results.

## 1. Introduction

Paraneoplastic neurological syndrome (PNS) is an immune-mediated neurological disorder caused by antibodies against intracellular, neuronal surface or synaptic proteins expressed by cancer cells. The detection of these antibodies is useful for PNS diagnosis and recent studies report the presence of a variety of onconeural antibodies (1).

Amphiphysin (AMPH), an intracellular synaptic vesicle protein, plays a critical role in regulating clathrin-coated synaptic vesicles (2). Anti-AMPH antibodies were initially reported in three women with paraneoplastic stiff-person syndrome (SPS) by De Camilli et al. (3). However, several reports and studies have demonstrated that AMPH autoimmunity is related to a broad spectrum of neurological manifestations, such as limbic encephalitis, peripheral neuropathy, myelopathy, brainstem encephalitis, and cerebellar dysfunction (4–9). Two case series studies have reported that the most common neurological manifestations are limbic encephalitis and neuropathy (7, 9). Furthermore, only < 10% of anti-AMPH antibody-positive patients fulfill the diagnostic criteria for SPS (9).

Herein, we report a case of anti-AMPH syndrome in a patient with gastric adenocarcinoma who presented with semi-rapidly progressive systemic tremors and rigidities followed by encephalopathy and peripheral neuropathy. Despite the negative results of fixed tissue-based assay (TBA) in detecting antineuronal antibodies, the presence of anti-AMPH antibodies was suspected in commercial immunoblots, which was subsequently confirmed by immunoprecipitation assay. The patient's symptoms were effectively treated with a combination of high-dose methylprednisolone, intravenous immunoglobulin (IVIg), and tumor resection, resulting in significant improvement in cognitive impairment and surprising recovery of the diffuse cerebral atrophy (DCA).

## 2. Methods

A range of surface and intracellular antineuronal antibodies were analyzed using TBA, as previously reported (10, 11). Briefly, adult female Wistar rats were sacrificed without perfusion and their brains were removed and fixed in 4% paraformaldehyde for 1 h at 4°C. The brains were cryoprotected in 40% sucrose for 48 h, embedded in freezing compound media, and snap-frozen in isopentane chilled with liquid nitrogen. Subsequently, 6-μm-thick tissue sections were incubated with 0.3% hydrogen peroxide for 15 min, 5% goat serum for 1 h, and patient and control sera (1:200) at 4°C overnight. After incubating with biotinylated secondary antibodies against human IgG (1:2,000, BA-3000, Vector), immunoreactivity was developed using the avidin–biotin–peroxidase method. Two experts (M.H. and H.N.) who are familiar with the immunohistochemical technique independently evaluated the assay results.

Immunoprecipitation was performed using an extract from HEK293T cells expressing FLAG-tagged human AMPH. The cells were lysed in the lysis buffer (TNET: 1M Tris-HCl pH 7.5, 450 mM NaCl, 0.5 M EDTA, 10% Triton X-100, protease inhibitors). The extract was pre-cleared with 20 μL of protein G Sepharose beads (GE Healthcare, Chicago, Illinois, IL, U.S.A.) for 30 min at 4°C to avoid non-specific binding. Similarly, the same amount of IgG in the serum from a healthy control or the patient was immobilized in 15 μL of protein G Sepharose for 30 min at 4°C. After a brief washing, the pre-cleared extracts were incubated with the immobilized IgG for 30 min at 4°C, and then extensively washed with TNET buffer. The bound IgG and protein complex was eluted from the beads by boiling in 30 μL of a sodium dodecyl sulfate-polyacrylamide gel electrophoresis sample buffer. The eluents were subjected to sodium dodecyl sulfate-polyacrylamide gel electrophoresis and detected with anti-DDDDK-tag (FLAG) antibodies (MBL, Tokyo, Japan) or anti-human IgG-HRP antibodies (Proteintech, Rosemont, Illinois, IL, USA).

Statistical significance was evaluated using one-way analysis of variance and the *post hoc* Tukey's test, and statistical significance was set at *p* < 0.05.

## 3. Case description

A 75-year-old woman with a history of hypertension and dyslipidemia presented to our hospital with oral and bilateral upper limb tremors that had been worsening over the last 3 months. Her first clinical presentation was intractable oral tremor, which had appeared 8 months prior to her first visit.

**Figure 1:**
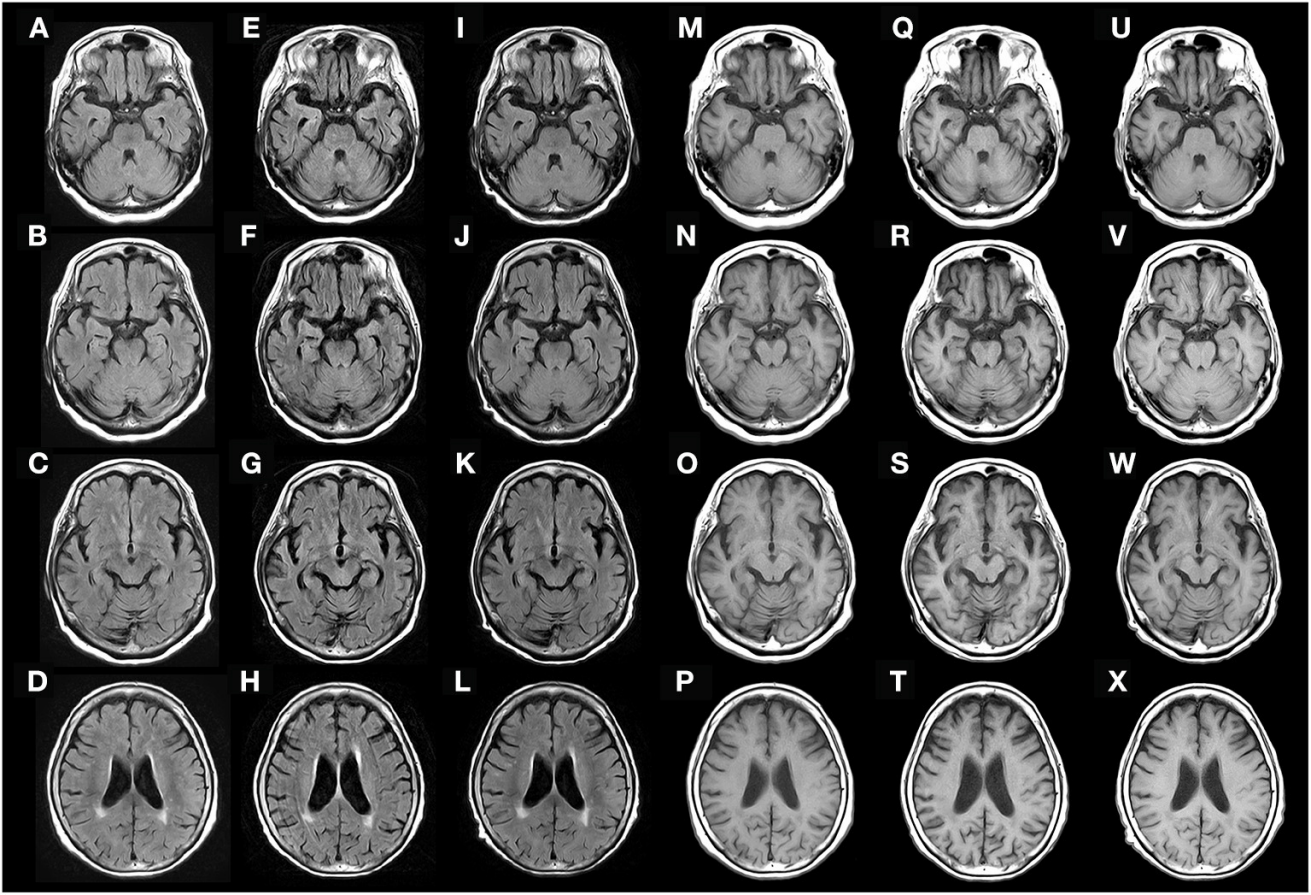
Fluid-attenuated inversion recovery (FLAIR) **(A–L)** and T1-weighted **(M–X)** brain MRI obtained 3 months before admission **(A–D, M–P)**, on admission **(E–H, Q–T)**, and 9 weeks after the initial treatment **(I–L, U–X)**. Diffuse cerebral atrophy was observed on admission **(E–H, Q–T)**, but rapidly recovered after immunotherapy **(I–L, U–X)**.

At the first visit, she exhibited oral tremor, asymmetric tremor, cogwheel rigidity, brachybasia, postural reflex disorder, and constipation, which were not contradictory to Parkinson's disease (PD). Levodopa/decarboxylase inhibitors were prescribed, which resulted in a slight improvement in her parkinsonism-like syndrome (tremors and rigidities), but not significantly. Two months later, she complained of visual hallucinations. Brain magnetic resonance imaging (MRI) did not show clear abnormal intensities or specific atrophy (Figures 1A–D, M–P). Therefore, we suspect rapidly progressive PD and subsequent dementia with Lewy bodies (DLB), and prescribed donepezil; however, the hallucinations did not improve. The following month, her systemic tremor and visual hallucinations worsened, and irritability increased despite medication use. She was admitted to our hospital for careful examination 3 months after her first visit.

On admission, her vital signs were as follows: body temperature, 37.5°C; heart rate, 80 bpm; and blood pressure, 151/98 mmHg. Her oxygen saturation level was 97% at ambient air.

Neurological examination confirmed disturbance of consciousness (Glasgow Coma Scale score of 13 [E4V4M5]) with severe restlessness, visual hallucination, irritability, cognitive impairment [Hasegawa's Dementia Scale-Revised (HDS-R) score of 6 out of 30], decreased deep tendon reflexes in the lower limbs, severe systemic tremor mainly in the limbs, and asymmetric cog-wheel rigidity in the upper and lower limbs. Due to the irritability and severe tremor, we could not evaluate her neurological symptoms related to ataxia. Moreover, sensory disturbance and limb numbness could not be confirmed. She was unable to stand on admission.

Initial laboratory tests did not reveal any specific abnormalities. Complete blood count, liver function, and renal function test results were assessed to be within reference range (RR) despite the mildly increased C-reactive protein level (2.97 mg/dL; RR: < 0.30 mg/dL). Tumor-specific laboratory tests revealed high serum levels of tumor markers (carcinoembryonic antigen, 6.06 ng/mL; cancer antigen 125, 132.5 ng/mL). The RR of CEA is < 5.0 ng/mL, and CA125 is < 35 ng/mL, respectively. Cerebrospinal fluid analyses showed elevated levels of lactate dehydrogenase (LDH) (42 IU/L; RR: < 25 IU/L) and interleukin-6 (IL-6) (11.3 pg/mL; RR: < 4.0 pg/mL) with normal cell counts (two leucocytes/μL) and total protein levels (43 mg/dL; RR: 10–50 mg/dL). Oligoclonal bands were negative. Serum paraneoplastic screening by fixed TBA did not show any significances (Supplementary Figure 1); however, EUROLINE PNS 12 Ag (Euroimmun) showed a weak positive 6 and 10, suggesting a low titer of anti-AMPH antibodies.

Brain MRI revealed semi-rapidly progressive DCA over a period of 3 months without clear abnormal intensities (Figures 1E–H, Q–T). Contrast-enhanced brain MRI was also performed, but no clear abnormal gadolinium enhancement was observed (data not shown). Contrast-enhanced whole-body computed tomography (CT) did not reveal any tumors, and no epileptic discharges were observed on electroencephalography. A nerve conduction study was not performed before treatment, and radionucleotide testing, such as positron emission tomography-CT, single-photon emission CT, dopamine transporter scan, and ^123^I-metaiodobenzylguanidine myocardial scintigraphy, could not be performed because it was not available in our hospital.

Based on her symptoms, which did not contradict encephalopathy, and the possible detection of anti-AMPH antibodies, we suspected that she had anti-AMPH syndrome. She received two courses of high-dose methylprednisolone over 2 weeks (1,000 mg/day × 3 days intravenously as one course per week). After treatment, her fever and visual hallucinations resolved completely, and her systemic tremor slightly improved, but remained moderate. At that time, she could stand using an assisting device, but could not walk. However, after initiating intravenous immunoglobulin (IVIg) treatment (4 weeks after the initial high-dose methylprednisolone therapy), she could walk without the use of any assisting devices, and her cognitive impairment partially recovered (HDS-R score of 22). At that time, the abnormal increase in LDH and IL-6 in the cerebrospinal fluid had decreased (LDH, 22 IU/L; IL-6, 2.6 pg/mL). Over the next 5 weeks (9 weeks after initial treatment), her cognitive impairment had almost fully resolved (HDS-R score of 28) and DCA had partially recovered (Figures 1I–L, U–X). To determine the presence of peripheral neuropathy, we performed a nerve conduction study after the IVIg treatment, which showed low-amplitude compound muscle action potential and sensory nerve action potential, suggesting axon loss rather than demyelination (data not shown).

**Figure 2:**
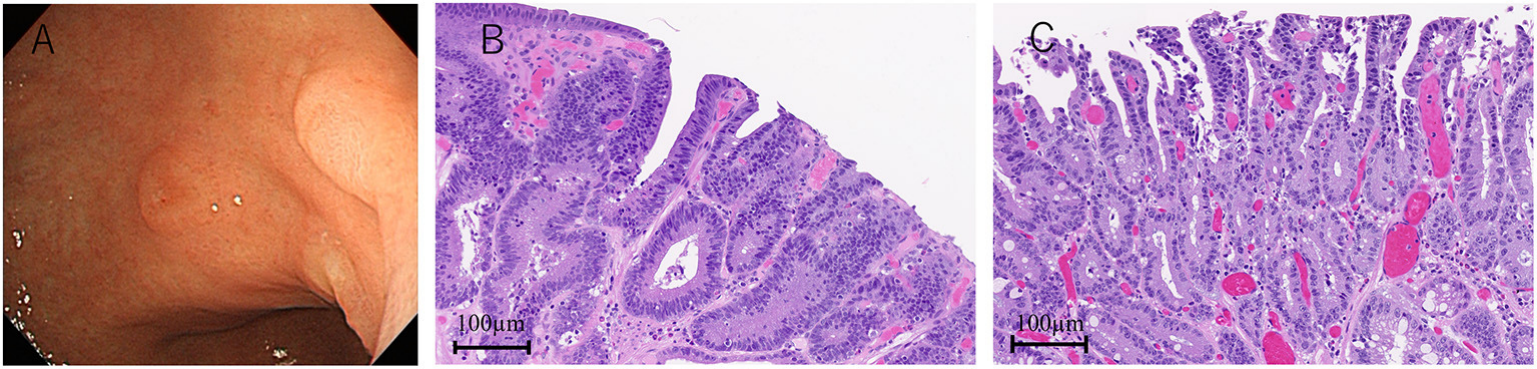
Gastroscopy and pathological findings. Two lesions of type 0-IIa gastric tumor **(A)**. Hematoxylin-eosin staining shows two adenocarcinomas **(B, C)**.

Because the anti-AMPH antibody is known as a paraneoplastic antineuronal antibody, we performed various tests to detect malignancy. Although colonoscopy and random skin biopsy showed normal findings, gastroscopy revealed type 0-IIa gastric tumors, and histological results suggested gastric adenocarcinomas with no evidence of metastasis (Figure 2A). After IVIg treatment (6 weeks after the initial high-dose methylprednisolone therapy), endoscopic submucosal dissection was performed and the tumors were completely removed. The pathological diagnosis was two well-differentiated adenocarcinoma lesions (Figures 2B, C).

**Figure 3:**
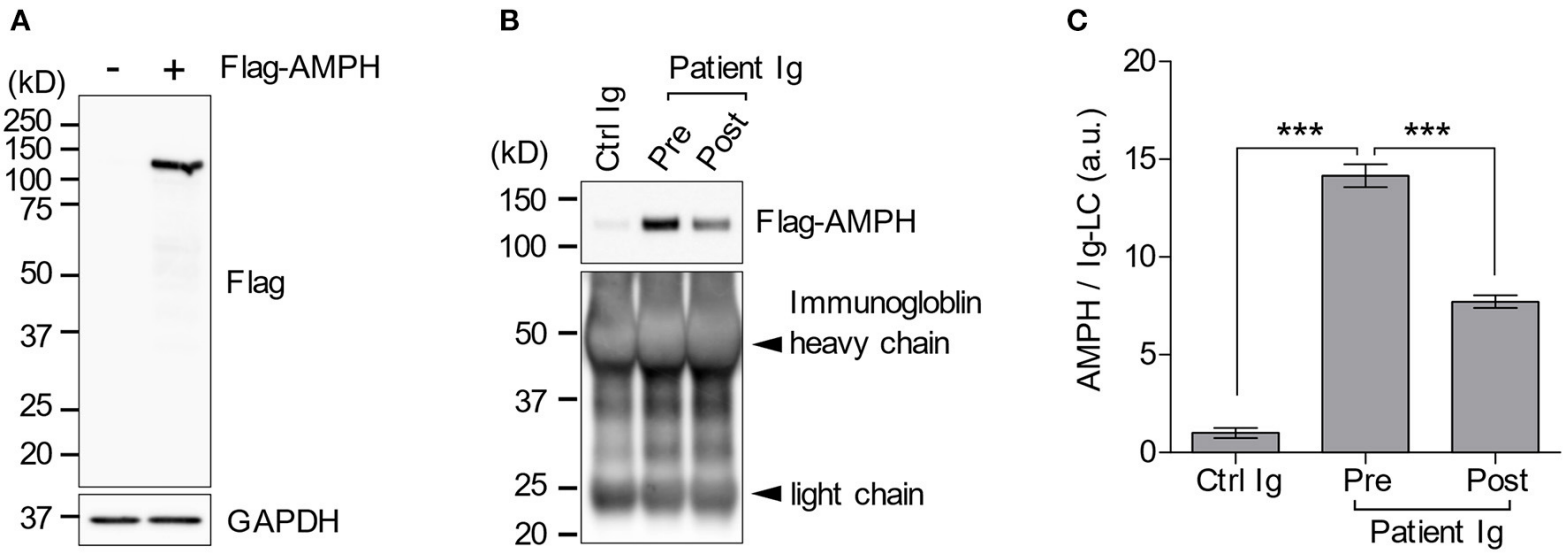
Immunoprecipitation assay. Flag-AMPH combined protein was successfully expressed in HEK-293T cells. However, the anti-FLAG antibody did not show any non-specific bands **(A)**. The patient's serum contained anti-AMPH antibodies, which decreased after immunotherapy **(B, C)**. AMPH, amphiphysin; FLAG, anti-DDDDK-tag; GAPDH, glyceraldehyde-3-phosphate dehydrogenase; Ig, immunoglobulin. ***p < 0.0001.

Despite active immunotherapy and tumor resection, her oral tremor persisted at a moderate degree. Although, the oral intake of baclofen slightly improved it, she did not experience a full recovery. Nonetheless, she could walk independently without difficulty. The patient was discharged 74 days after admission. We also performed an immunoprecipitation assay since TBA failed to detect anti-AMPH antibodies in the patient's serum. A subsequent western blotting analysis revealed the presence of anti-AMPH antibodies and their continuous decrease 10 months after immunotherapy (Figures 3A–C). The clinical timeline is shown in Supplementary Figure 2.

## 4. Discussion

In this report, we presented a rare case of reversible brain atrophy in anti-AMPH syndrome in a patient with gastric adenocarcinoma who presented with semi-rapidly progressive systemic tremors, rigidities, encephalopathy, peripheral neuropathy, and DCA. The symptoms were markedly reduced by active immunotherapy and tumor resection. As all treatments were applied within a relatively short time period of each other and were effective, it was difficult to determine which was the most effective treatment.

In the early stage, the diagnostic criteria advocated by the Internal Parkinson and Movement Disorder Society in 2015 (12), this patient's symptoms were consistent with “clinically probable PD” because we could not find any “absolute exclusion criteria” or “red flags.” In addition, the manifestation of visual hallucinations suggested co-occurrence of DLB. However, on admission, the patient showed rapidly progressive gait impairment requiring regular use of a wheelchair within 1 year of onset. This was a “red flag” for PD. Finally, the detection of anti-AMPH antibodies ruled out the diagnosis of PD/DLB. Moreover, the possibility of progressive supranuclear palsy or multiple system atrophy had also been ruled out.

The latest diagnostic criteria of paraneoplastic neurological syndrome (PNS) were published in 2021 (13). To meet the criteria for “definite PNS,” confirmation of the cancer typically associated with the detected antibody is necessary. In the case of anti-AMPH antibodies, breast or small cell lung cancer is usually associated. If the cancer is not typical for the detected antibody, the diagnosis of “definite PNS” requires demonstrating antigen expression by the tumor. Our patient met only the diagnostic criterion of “probable PNS” because gastric adenocarcinoma is not typical in anti-AMPH syndrome and the expression of antigen on the cancer was not tested. Moreover, the clinical findings in our patient were atypical of autoimmune encephalitis due to long-term progression (>3 months), the absence of cerebrospinal fluid pleocytosis, and MRI features suggestive of encephalitis (14); however, the patient exhibited cognitive impairment, visual hallucinations, and irritability. Therefore, we diagnosed the patient with encephalopathy related to anti-AMPH antibodies.

Although the reason why fixed TBA failed to detect serum antibodies is not clear, it is widely recognized not all antibodies that work in immunoprecipitation are equally adaptable for use in immunohistochemistry. Several factors, such as antibody titer, antigen-specificity, and epitope masking during fixation, can influence the outcome of immunohistochemistry experiments. One possible reason could be that the concentration of anti-AMPH antibodies was very low to be detected by fixed TBA. Moreover, in this study, we used rat brain for fixed TBA, which may possess different epitopes compared to human AMPH. Furthermore, the low sensitivity of fixed TBA has already been reported in the other autoimmune diseases (15, 16). In a recent study, commercial immunoblots have been reported to show high rates of false positivity, and none of the weak-positive sera in EUROLINE PNS 12 Ag were confirmed by other techniques (17). However, serum antibody purification was not performed, and false negatives could not be completely ruled out, particularly in low-titer anti-AMPH antibody cases. Nonetheless, further studies are needed to determine the sensitivity and specificity of immunoprecipitation for PNS-related autoantibodies, because this is the first report of detecting these autoantibodies by immunoprecipitation.

AMPH is classified as an intracellular synaptic antigen, which distinguishes it from typical intracellular antigens found in the nucleus and cytoplasm (18, 19). Unlike typical antibodies against intracellular antigens, anti-AMPH antibody may be directly pathogenic, as demonstrated by the passive transfer of purified anti-AMPH antibodies from patients to a rat, which resulted in SPS-like symptoms (tremors and rigidities). These antibodies may also induce synaptic dysfunction (gamma-aminobutyric acid release) and internalization of complexes into the cytoplasm (2, 20), suggesting a potential mechanism for their pathogenicity. In addition to SPS, AMPH autoimmunity has been associated with various neurological manifestations, including encephalitis and peripheral neuropathy, and various types of malignancies, including breast cancer, small-cell lung carcinoma, and gastric cancer (7, 9). Moon et al. (7) initially introduced the term “anti-AMPH syndrome” in 2014 to describe 20 anti-AMPH antibody-positive patients who did not meet the criteria for SPS, and found that IVIg and steroid treatments were effective in improving their symptoms (7). As described in the case presentation, our patient exhibited both encephalopathy and peripheral neuropathy, as well as tremors and rigidities.

Some patients were reported to be resistant to IVIg or steroid treatments; in those cases, second-line treatments, including rituximab or cyclophosphamide, would be considered (7, 21). To the best of our knowledge, no previous reports have described reversible brain atrophy in anti-AMPH syndrome. However, a few toxic, metabolic, and endocrine disorders have been reported to cause reversible brain atrophy (22–24). Among autoimmune encephalitis cases, only those with anti-N-methyl-D-aspartate receptor (NMDAR) encephalitis have been reported to show reversible DCA (25–28). Iizuka et al. initially reported two young women who presented with reversible DCA and anti-NMDAR encephalitis (25). They also reported that DCA might occur in about 33% of anti-NMDAR encephalitis cases, but was not associated with poor outcomes unless cerebellar atrophy coexisted (29). Regarding the mechanisms of DCA, they speculated the following risk factors: systemic complications (heart failure and septic shock), status epilepticus, malnutrition, prolonged use of corticosteroids, long-term exposure to various antiepileptic agents, and prolonged use of propofol (29). However, at the time DCA was observed, our patient was not treated with corticosteroids, antiepileptic agents, or propofol. Systemic complications (heart failure or septic shock) and malnutrition were not observed. In addition, electroencephalography did not detect epileptic discharges. Thus, the postulated mechanisms were not applicable to our patient. Still, one possible mechanism of DCA is explained by NMDAR internalization and its dysfunction without neuronal death (29). Although the mechanism underlying the recovery from brain atrophy in our case remains unclear, our findings suggest that early active immunotherapies, combined with tumor resection and low antibody titers, can contribute to favorable outcomes in the recovery of brain atrophy.

## 5. Conclusion

In conclusion, we reported a case of anti-AMPH syndrome in a patient with gastric adenocarcinoma who presented with semi-rapidly progressive systemic tremors followed by encephalopathy and peripheral neuropathy. The patient showed a significant reduction in symptoms following active immunotherapies and tumor resection. Early diagnosis and treatment may lead to improved clinical outcomes in patients with anti-AMPH syndrome. Furthermore, it should be noted that negative TBA results with positive commercial immunoblots do not necessarily indicate a false positive, particularly in case with low-titer antineuronal antibodies.

## Data availability statement

The original contributions presented in the study are included in the article/Supplementary material, further inquiries can be directed to the corresponding author.

## Ethics statement

The studies involving human participants were reviewed and approved by the Ethics Review Board of Fujimi Kogen Hospital (Reference Number 126). The patients/participants provided their written informed consent to participate in this study. Written informed consent was obtained from the individual(s) for the publication of any potentially identifiable images or data included in this article. Written informed consent was obtained from the participant/patient(s) for the publication of this case report.

## Author contributions

RA was the attending physician, decided on the treatment policy, collected patient data, and performed immunoprecipitation. Y-JK prepared the recombinant plasmid, transfected cells, and performed western blotting analysis. TY and MY examined the patient and provided a critical opinion regarding encephalopathy. MH and HN performed and evaluated the TBA. RA played a major role in study conception and drafted the manuscript. TO revised the manuscript for important intellectual content. All authors have read and approved the final version of the manuscript.
