# Supplementary material for: Case report: Reversible brain atrophy with low titer anti-amphiphysin antibodies related to gastric adenocarcinoma
Source: Front Neurol. 2023 Jun 21;14:1211814. doi: 10.3389/fneur.2023.1211814 (PMC10322512; doi:10.3389/fneur.2023.1211814)
Supplement: Supplementary file 1 [file Data_Sheet_1.docx]

Supplementary Material

# Supplementary Figure 1


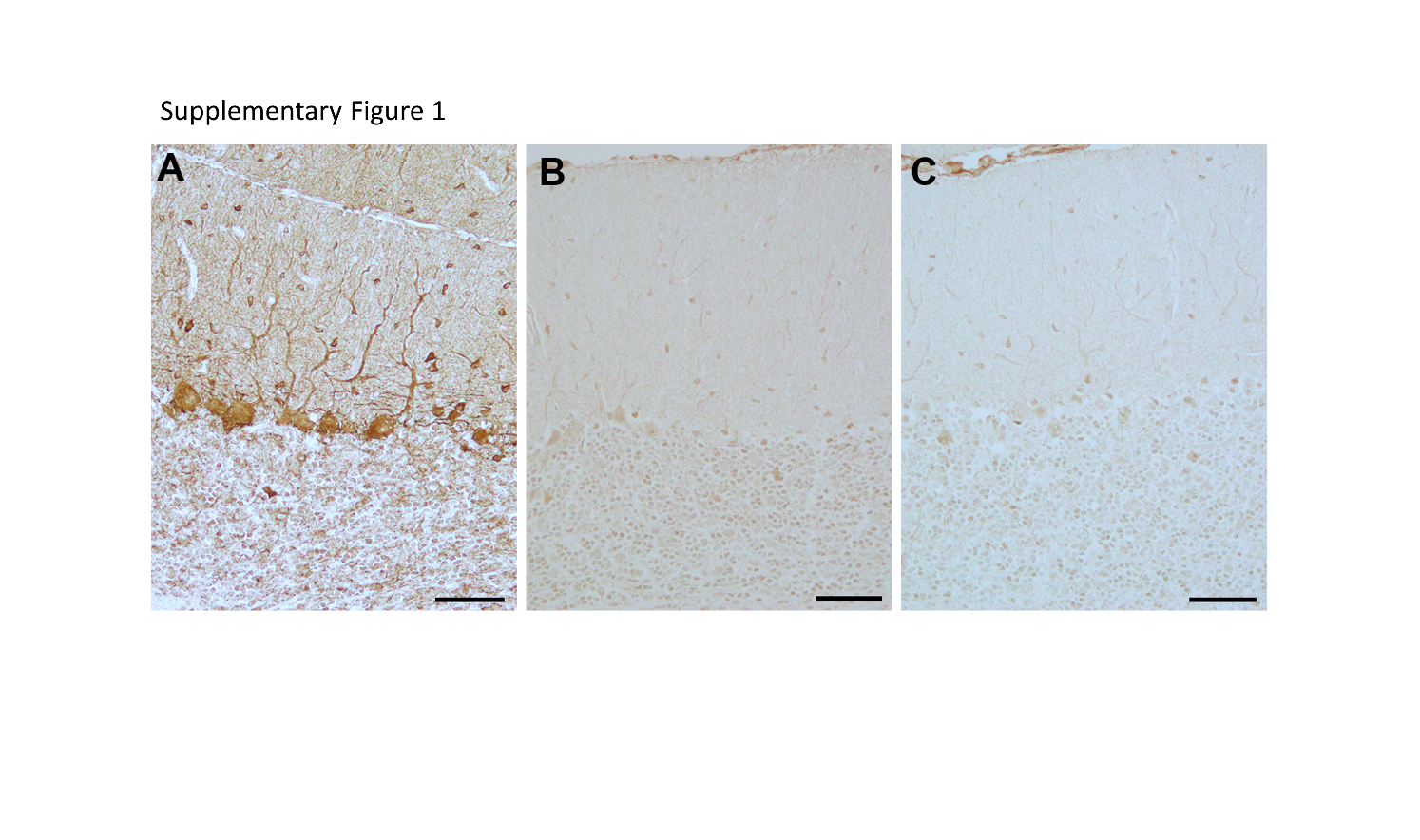


**Supplementary Figure 1.** In-house indirect immunohistochemistry (IHC) using rat cerebellar sections permeabilized with 0.5% Triton X-100 for screening of autoantibodies against intracellular antigens.

IHC was implemented on rat cerebellar sections for control (A, B) and patient’s serum (C) samples (C). Note that a control serum that was proven to be glutamic acid decarboxylase 65 positive (A) labeled the synapses of Purkinje cells and stratum granulosum in the cerebellar cortex. In contrast, the rat cerebellum was not labeled by either the negative control (B) or the patient's (C) serum sample. In-house IHC results revealed no evidence of autoantibodies against paraneoplastic intracellular antigens that included amphiphysin in the patient’s serum sample. All bars indicate 50 µm.

# Supplementary Figure 2


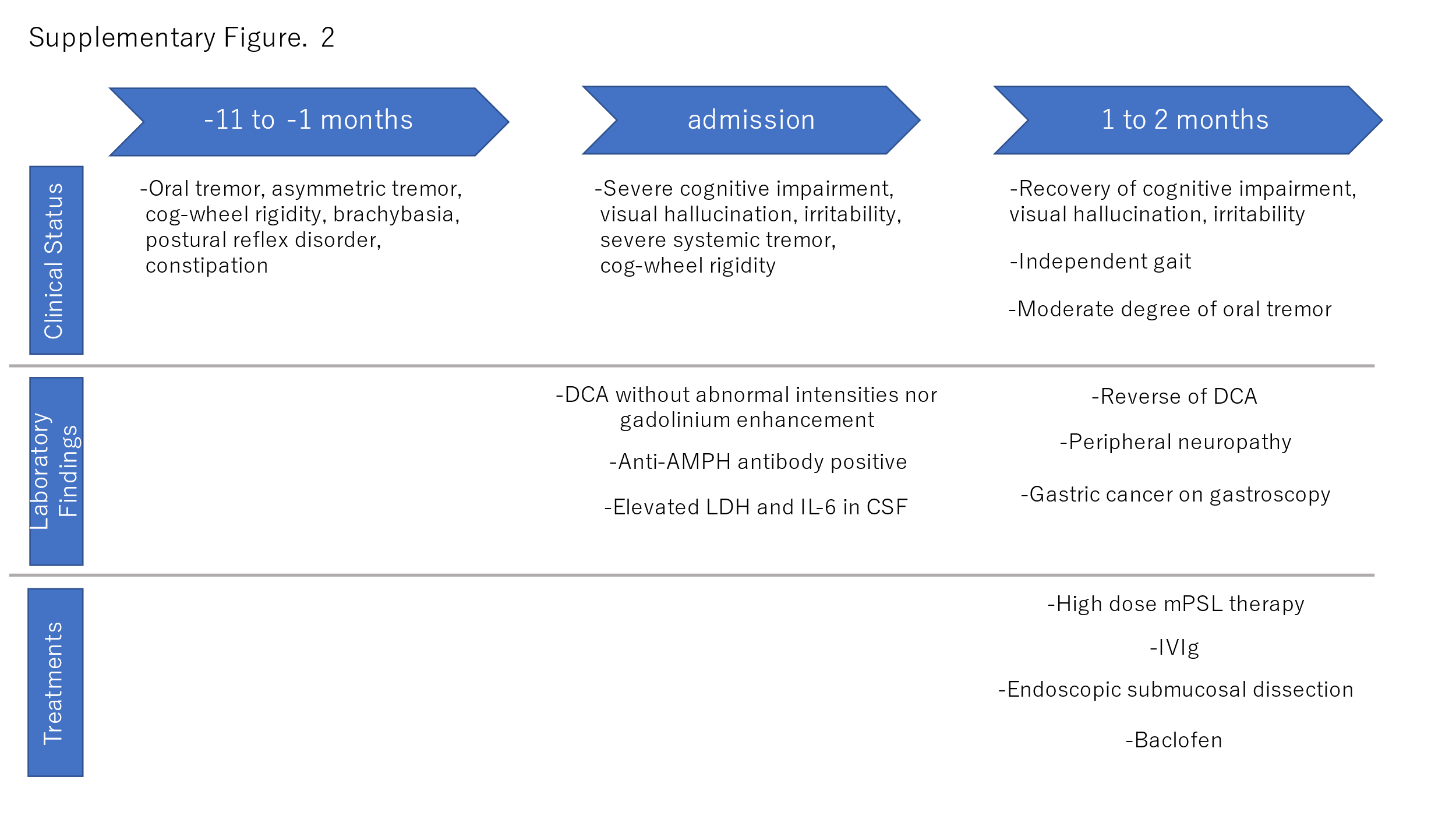


**Supplementary Figure 2.** Clinical timeline. DCA, diffuse cerebral atrophy; AMPH, amphiphysin; TBA, tissue-based assay; LDH, lactate dehydrogenase; IL-6, interleukin-6; CSF, cerebrospinal fluid; mPSL, methylprednisolone; IVIg, intravenous immunoglobulin.
